# Carnitine promotes recovery from oxidative stress and extends lifespan in *C. elegans*

**DOI:** 10.18632/aging.202187

**Published:** 2020-12-03

**Authors:** Dongliang Liu, Xiaofang Zeng, Le Li, Zheng-Lin Ou

**Affiliations:** 1 Department of Spine Surgery, Xiangya Hospital, Central South University, Changsha 410008, China; 2 Department of Cardiology, Xiangya Hospital, Central South University, Changsha 410008, China; 3 Hunan Yuantai Biotechnology Co., Ltd, Changsha 410000, Hunan, China; 4 Department of General Surgery, Xiangya Hospital, Central South University, Changsha 410008, China

**Keywords:** carnitine, aging, oxidative stress, amyloid, transporter

## Abstract

Carnitine is required for transporting fatty acids into the mitochondria for β-oxidation. Carnitine has been used as an energy supplement but the roles in improving health and delaying aging remain unclear. Here we show in *C. elegans* that L-carnitine improves recovery from oxidative stress and extends lifespan. L-carnitine promotes recovery from oxidative stress induced by paraquat or juglone and improves mobility and survival in response to H_2_O_2_ and human amyloid (Aβ) toxicity. L-carnitine also alleviates the oxidative stress during aging, resulting in moderate but significant lifespan extension, which was dependent on SKN-1 and DAF-16. Long-lived worms with germline loss (*glp-1*) or reduced insulin receptor activity (*daf-2)* recover from aging-associated oxidative stress faster than wild-type controls and their long lifespans were not further increased by L-carnitine. A new gene, T08B1.1, aligned to a known carnitine transporter OCTN1 in humans, is required for L-carnitine uptake in *C. elegans*. T08B1.1 expression is elevated in *daf-2* and *glp-1* mutants and its knockdown prevents L-carnitine from improving oxidative stress recovery and prolonging lifespan. Together, our study suggests an important role of L-carnitine in oxidative stress recovery that might be important for healthy aging in humans.

## INTRODUCTION

Carnitine, a water-soluble quaternary amine (3-hydroxy-4-N, N, N-trimethyl amino butyric acid), is synthesized from the essential amino acids lysine and methionine [1, 2]. Cells utilize L-carnitine for transporting long chain fatty acids into mitochondria for β-oxidation [3]. L-carnitine is essential for lipid metabolism in all cell types and highly enriched in skeletal and cardiac muscle [4]. Various forms of carnitine, including L-carnitine, acetyl-L-carnitine, and propionyl-L-carnitine, have long been used as dietary supplements for weight loss and enhancing performance [5]. Impaired carnitine uptake due to genetic mutations in the carnitine-transporters can cause cardiomyopathy, skeletal-muscle weakness, and hypoglycemia [6–8]. Certain conditions such as chronic renal failure and antibiotics abuse can also reduce carnitine retention in the body, manifesting symptoms of carnitine deficiency [8, 9]. Carnitine has been approved by the Food and Drug Administration (FDA) to treat primary and certain secondary carnitine-deficiency syndromes [10].

Carnitine levels decrease during aging, which may contribute to the decline in mitochondrial function and aging [11]. In rats, dietary supplementation with high doses of acetyl-L-carnitine and alpha-lipoic acid improves mitochondrial function and decreases oxidative stress, resulting in improved performance on memory-requiring tasks [12–14]. In humans, several studies suggest that carnitine could improve mental health in older adults with mild cognitive impairment or Alzheimer’s disease [15]. Carnitine may also improve other age-associated diseases such as cardiovascular diseases [16–19], atherosclerosis [20], neurometabolic disorders [21] and diabetic symptoms [22–25]. In addition, carnitine has also been used in adjuvant therapy to treat conditions such ashemodialysis [26]. Acetyl-L-carnitine can extend chronological lifespan of budding yeast [27, 28]. Most of the therapeutic effect of carnitine have been linked to cellular redox balance [29, 30]. However, the detailed mechanisms of action at the cellular and molecular levels have not been fully investigated.

The round worms *C. elegans* has been widely used to study oxidative stress response and aging [31, 32]. In response to chemicals that generate reactive oxygen species (ROS) such as paraquat or juglone, *C. elegans* activates SKN-1 and DAF-16 dependent transcription programs to alleviate oxidative damage, which leads to lifespan extension [33, 34]. Activation of the SKN-1 pathway contributes to the extended lifespan of *glp-1* mutant lacking germline and *daf-2* mutant with impaired insulin receptor [35, 36]. The roles of SKN-1 and DAF-16 are conserved in mammals (Nrf2 and FOXO3, respectively), providing useful targets for disease intervention [37]. *C. elegans* has also been used for study human Aβ toxicity, in which ectopic expression of human Aβ causes paralysis [38]. Very few studies have been carried out in *C. elegans* regarding carnitine metabolism and function. In one study, L-carnitine supplement in the medium can prevent glucose toxicity on *C. elegans* survival [39]. In another study, feeding worms with acetyl-carnitine reduces age-related neuronal damage and improves learning behavior [40]. Whether carnitine has a role in OSR and aging in *C. elegans* has not been reported.

In this study, we show that carnitine supplement in the medium facilitates recovery from oxidative stress during aging, promoting resistance to oxidative toxicity induced by H_2_O_2_ and human amyloid protein Aβ(1–42) aggregates. Carnitine’s beneficial effect is dependent on SKN-1, DAF-16 and a potential carnitine transporter T08B1.1. T08B1.1 expression is increased in the longevity mutant *glp-1* and required for the long lifespan. Our study is the first of its kind to report the effect of carnitine on aging in *C. elegans* and reveal novel roles of carnitine in extending lifespan.

## RESULTS

### L-carnitine shortens the length of oxidative stress response (OSR)

**Figure 1:**
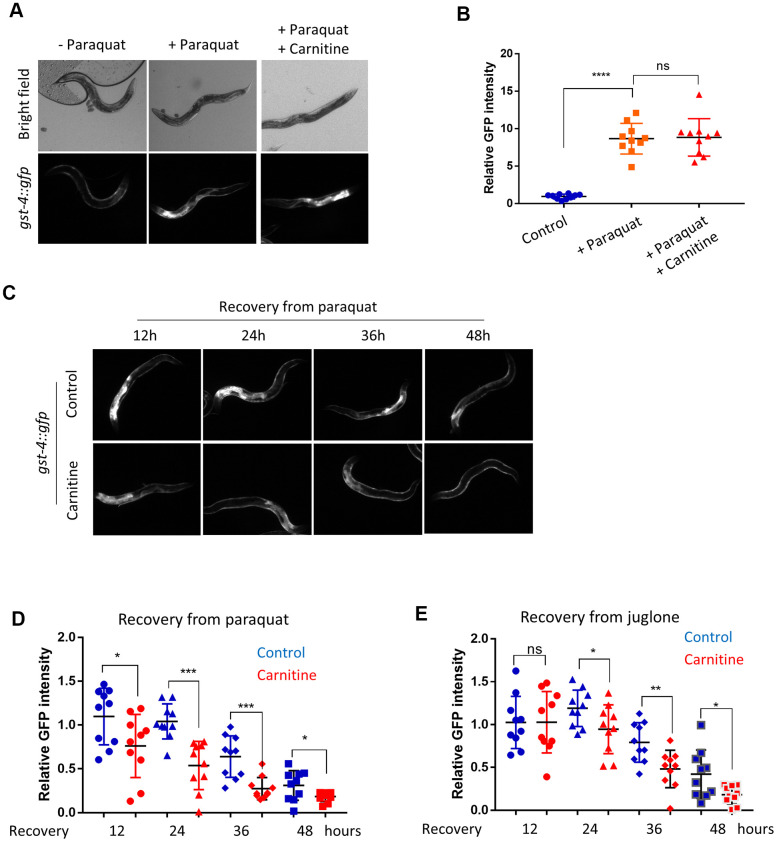
**L-carnitine shortens the length of oxidative stress response (OSR) induced by paraquat and juglone.** (**A**) L-carnitine did not affect induction of OSR. *C. elegans* expressing the OSR marker *gst-4::gfp* were synchronized at L1 larvae stage and raised on NG medium supplemented with or without 10 μM L-carnitine to L4/young adult stage. The ROS generator paraquat was then added to the medium to the final concentration of 1 mM. After 24 hours, animals were imaged with a fluorescent microscope. Representative images were shown. (**B**) Quantification of images taken from at least 3 independent experiments in (**A**) by ImageJ and relative expression levels were plotted. Statistical analysis was performed by two-tailed, unpaired student’s t-test (ns, not significant. ****, P<0.0001). Error bars indicate the standard deviation of the mean. (**C**) L-carnitine reduced the *gst-4::gfp* expression during recovery from oxidative stress. *C. elegans* worms were prepared and treated as in (**A**). After 24 hours of paraquat treatment, worms were transferred to fresh plate. OSR marker *gst-4::gfp* were examined at indicated time. (**D**) Quantification of *gst-4::gfp* expression in (**C**). Images from 3 independent experiments were quantified using ImageJ and normalized to the value at time 0. Statistical analysis was performed by two-tailed, unpaired student’s t-test (*, P<0.05. ***, P<0.001). Error bars indicate standard deviation of the mean. (**E**) L-carnitine reduced *gst-4::gfp* expression during recovery from juglone treatment. *C. elegans* worms were prepared and treated as in (**C**) except that 300 μM juglone (final concentration) was added to the NG medium. Images from 2 independent experiments were quantified using ImageJ and normalized to the average at 12 hours. Statistical analysis was performed by two-tailed, unpaired student’s t-test (ns, not significant. *, P<0.05. **, P<0.001). Error bars indicate standard deviation of the mean.

It has been shown that L-carnitine has antioxidant activity *in vitro* [41]. By using paraquat, a well-known ROS generator affecting mitochondrial respiration and dynamics [42, 43], we tested if L-carnitine would have antioxidant activity *in vivo* in *C. elegans*. We raised the animals on agar plate containing nematode growth (NG) medium supplemented with and without 100 μM of L-carnitine from L1 stage to L4 stage, then oxidatively stressed the animals with 1mM paraquat for 24 hours on NGM plate. For convenience, we examined the OSR marker *gst-4::gfp* [44]. We found strong activation of *gst-4:gfp* by paraquat treatment, indicating a robust oxidative stress condition. L-carnitine supplementation did not significant affect *gst-4:gfp* induction by paraquat (Figure 1A, 1B). However, animals treated with L-carnitine had a better recovery from paraquat, as shown by a faster clearance of the OSR marker *gst-4::gfp* after transferring to paraquat-free medium (Figure 1C, 1D). To confirm these results, we also used another ROS generating compound juglone [45]. Similarly, *gst-4::gfp* levels decreased faster in animals treated with L-carnitine compared with non-treated animals (Figure 1E), suggesting that L-carnitine promotes oxidative stress recovery in *C. elegans*.

### L-carnitine promotes the recovery from oxidative stress

**Figure 2:**
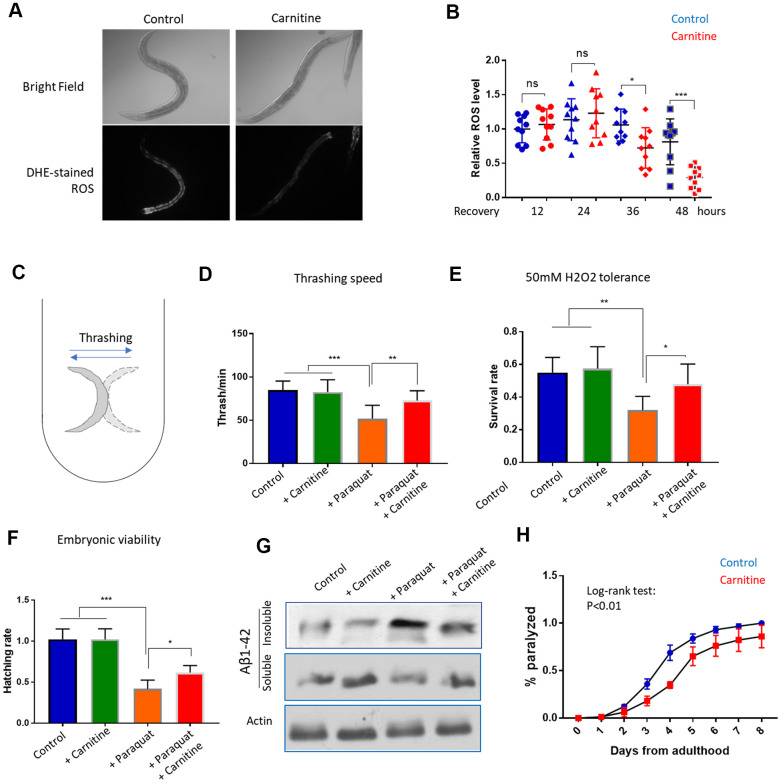
**L-carnitine promotes recovery from oxidative stress induced by paraquat and juglone.** (**A**) L-carnitine facilitated the clearance of ROS. N2 wild-type *C. elegans* were synchronized at L1 larvae stage and raised on NG medium supplemented with or without 10 μM L-carnitine to L4/young adult stage. The ROS generator paraquat was added to the medium to the final concentration of 1mM. After 24 hours, animals were transferred to new paraquat-free plate with and without L-carnitine for recovery. After recovery for 12, 24, 48 hours, worms were stained with ROS dye dihydroethidium (DHE) and imaged with fluorescent microscope. Representative images at 48-hour recovery were shown. (**B**) Quantification of DHE signal from at least 3 independent experiments in (**A**) by ImageJ and relative expression levels were plotted. Statistical analysis was performed by two-tailed, unpaired student’s t-test (ns, not significant. *, P<0.05. ***, P<0.001). Error bars indicate standard deviation of the mean. (**C**) Thrashing assay for *C. elegans*. Worms were pick from agar plate to 1 mL M9 buffer in 24-well plate and examined under dissecting microscope. Worms were moving left and right rapidly and the movement from one side to the other side then back to the original position was counted as 1 thrash. (**D**) L-carnitine mitigated the toxicity of paraquat on mobility. Worms were treated with paraquat and L-carnitine as in (**A**) and recovered for 48 hours. Trashing speed (thrash/min) under different treatments were measured for at least 3-independent experiments with 10 animals/experiment. Statistical analysis was performed by two-tailed, paired student’s t-test (**, P<0.01. ***, P<0.001). Error bars indicate standard deviation of the mean. (**E**) L-carnitine rescued the H_2_O_2_ hypersensitivity of paraquat-treated worms. Worms were treated with paraquat and L-carnitine as in (**A**) and recovered for 48 hours. Worms were then incubated in 50mM H_2_O_2_ for 1 hour. Data were pooled from 2 independent experiments and survival rates under different treatment were compared. Statistical analysis was performed by two-tailed, paired student’s t-test (*, P<0.05. **, P<0.01). Error bars indicate standard deviation of the mean. (**F**) L-carnitine mitigated the toxicity of paraquat on embryonic survival. Worms were treated with paraquat and L-carnitine as in (**A**) and recovered for 48 hours.10 worms were transferred to a new agar plate to allow egg laying for 2 hours and total number of eggs were counted. Hatching were examined after 24 hours. Experiments were conducted 2 times with 5 replicates each time. Data were normalized to control group for comparison. Statistical analysis was performed by two-tailed, unpaired student’s t-test (**, P<0.01. ***, P<0.001). Error bars indicate standard deviation of the mean. (**G**) L-carnitine mitigated paraquat-induced amyloid protein aggregation. Worms expressing human amyloid protein fragment Aβ(1-42) in body wall muscle (CL2006) were treated with paraquat and L-carnitine as in (**A**) and recovered for 48 hours. Worms were then homogenized in high-salt RAB buffer. Soluble and insoluble fraction were analyzed by western blot using anti-Aβ antibody. Total lysate was analyzed by western using anti-actin antibody. Shown was representative results of 2 independent experiments. (**H**) L-carnitine mitigated Aβ(1-42)-induced paralysis. Animals were raised at 25° C to young adult stage (day-0) and examined every day thereafter for paralysis. Data pooled from 2 independent experiments (n>120) were plotted. Log-rank test was performed (P<0.01). Error bar indicated the standard deviation of the mean.

To further confirm the new finding that L-carnitine improves recovery from oxidative stress, we directly measured the ROS levels. Animals raised with and without 100 μM L-carnitine supplement from L1 stage to L4 stage were challenged with 1mM paraquat for 24 hours, then transferred to paraquat-free medium plate. After 12, 24, 36 and 48 hours, animals were stained with Dihydroethidium (DHE), a widely used ROS indicator [35, 46]. The results showed that L-carnitine treatment robustly reduced the ROS levels in the intestine after 36 and 48 hours of recovery from paraquat (Figure 2A, 2B).

Next, we tested multiple heath parameters related to oxidative stress. First, we examined if muscle strength could be improved by L-carnitine, by measuring the bending movement. When in liquid, the worms keep bending or thrashing (Figure 2C), which has been used to indicate the muscle strength and mobility [47]. By treating the animals with paraquat and L-carnitine as mentioned above, we measured the thrashing speed after 48 hours of recovery. The results showed that the impaired mobility by paraquat treatment could be largely rescued by L-carnitine (Figure 2D). L-carnitine did not obviously improve mobility under normal conditions. Second, we tested if the faster recovery from oxidative stress could result in a better tolerance to oxidative damage by H_2_O_2_. Similarly, animals treated with paraquat and L-carnitine were collected 48 hours after recovery from paraquat and incubated with 50mM of H_2_O_2_ for 1 hour. We found that paraquat-treated worms were sensitive to H_2_O_2_ toxicity, which was largely rescued by L-carnitine (Figure 2E). Third, we examined the survival rate of progenies. Consistently, egg hatching was reduced by paraquat, but such reduction was significantly mitigated by L-carnitine (Figure 2F). Fourth, we examined if human amyloid protein aggregation could be mitigated by supplementing L-carnitine in the medium. Amyloid-beta (Aβ1-42) has been known to aggregate upon oxidative stress or during aging [48, 49]. By separating the soluble and insoluble fractions of the whole worm lysate and western blotting, we found that L-carnitine reduced the paraquat-induced Aβ1-42 aggregation (Figure 2G). Interestingly, despite the lack of effect of L-carnitine on Aβ1-42 aggregates under normal culturing conditions (Figure 2G), the animals treated with L-carnitine slightly but significantly decreased amyloid-induced paralysis (Figure 2H). Together, these functional assays confirmed that L-carnitine reduced oxidative stress damage in *C. elegans*.

### L-carnitine decreases oxidative stress during aging and increased lifespan in *C. elegans*

**Figure 3:**
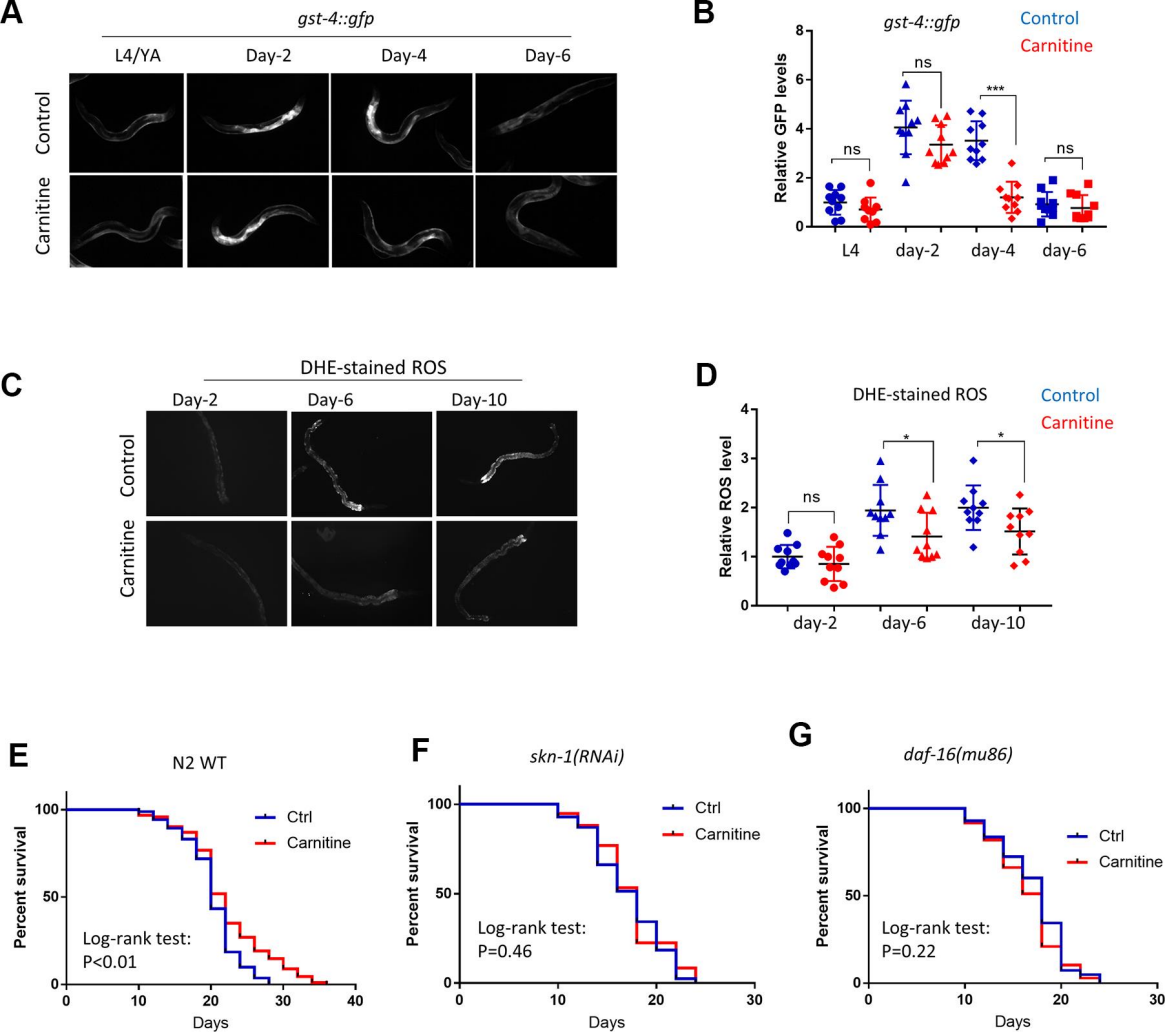
**L-carnitine promotes oxidative stress recovery during aging and increased lifespan in *C.elegans*.** (**A**) L-carnitine promoted recovery from oxidative stress during aging. *C. elegans* expressing the OSR marker *gst-4::gfp* were synchronized at L1 larvae stage and raised on NG medium supplemented with or without 10 μM L-carnitine. Worms were imaged with fluorescent microscope at L4/young adult (YA) stage, day-2, day-4 day-6 of adulthood. Representative images of 3 independent experiments were shown. (**B**) Quantification of images from experiment described in (**A**). Shown were *gst-4::gfp* levels normalized to the average of L4/young stage. Statistical analysis was performed by two tailed, unpaired student’s t-test (ns, not significant. ***, P<0.001). Error bars indicate standard deviation of the mean. (**C**) L-carnitine delayed the ROS accumulation in *C. elegans* during normal aging. Worms were treated as in (**A**) and stained with DHE dye at indicated time points, followed by imaging with fluorescent microscope. Representative images from 3 experiments were shown. (**D**) Quantification of DHE-stained ROS levels described in (**C**). Images from 3 experiments were quantified by ImageJ and statistically analyzed by two-tailed, unpaired student’s t-test (ns, not significant. *, P<0.05). Error bars indicate standard deviation of the mean. (**E**) L-carnitine prolonged the lifespan of wild-type N2 *C. elegans*. Wild-type *C. elegans* were synchronized at L1 larvae stage and raised on NG medium supplemented with or without 10 μM L-carnitine. 50 μM FUDR was added to prevent reproduction. Dead and viable worms were counted every 2 or 3 days starting from day-10 of adulthood. Experiments were performed for 2 times (n>120) and survival were analyzed by log-rank test (Supplementary Table 1). (**F**) L-carnitine did not extend lifespan of *C. elegans* with *skn-1* knockdown. Experiments were performed similar to (**E**) except RNAi bacteria was used. Data were pooled from 2 experiments (n>120) and analyzed by log-rank test (Supplementary Table 1). (**G**) L-carnitine did not extend lifespan of *C. elegans* with *daf-16* knockdown. Experiments were performed similar to (**E**) except RNAi bacteria was used. Data were pooled from 2 experiments (n>120) and analyzed with log-rank test (Supplementary Table 1).

Since ROS accumulates during aging, L-carnitine could also improve age-related oxidative damage. To test this, *C. elegans* were cultured from L1 stage on NG medium supplemented with or without 100 μM L-carnitine and *gst-4::gfp* expression was examined at L4, day-2, day-4 and day-6 of adulthoods. Interestingly, *gst-4::gfp* was induced after reaching adulthood but gradually declined thereafter, consistent with a previous report [35]. Similar to that of paraquat treatment, aging-induced *gst-4::gfp* expression was decreased by L-carnitine during the recovery stage but not the induction stage (Figure 3A, 3B). Despite the decline in *gst-4::gfp* expression after day-4, DHE-stained ROS continued to accumulate from day-2 to day-10. Importantly, worms raised on L-carnitine appeared to have less ROS on day-6 and day-10 of adulthood, suggesting a better recovery from oxidative stress during aging (Figure 3C, 3D).

We also tested if aging was delayed in *C. elegans* by supplementing L-carnitine in the medium. Animals were cultured under 20° C on NG medium containing various concentrations of L-carnitine throughout life. 50 μM 5’-Fluoro-2-deoxyuridine (FUDR) was added to inhibit progeny growth. The results showed that L-carnitine extended lifespan of *C. elegans* at 100, 200 and 500 but not 50 μM (Figure 3E and Supplementary Figure 1A). At 200 and 500 μM, L-carnitine causes slight developmental delay in some worms (Supplementary Figure 1B). We therefore chose 100 μM for all experiments in this study. SKN-1 and DAF-16 are transcription factors known to activate oxidative stress defensing programs and extend lifespan in *C. elegans* [50]. Both RNAi knockdown prevented L-carnitine from extending lifespan, suggesting that SKN-1 and DAF-16 are both needed for L-carnitine to extend lifespan in *C. elegans* (Figure 3F, 3G).

### The long-lived mutants *daf-2* and *glp-1* recover better from oxidative stress than wild-type controls

**Figure 4:**
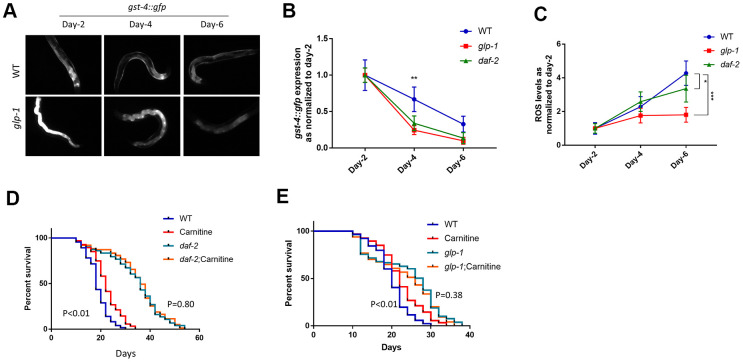
**The long-lived mutants *daf-2* and *glp-1* recover from oxidative stress better than wild-type controls.** (**A**) *glp-1* worms showed faster decrease in the expression of the OSR marker *gst-4::gfp*. Age-matched, *gst-4::gfp*-expressing WT and *glp-1* mutant worms were raised from L1 to L4 at 25° C to deplete germ cells in *glp-1* then maintained at 20° C throughout the experiment. *gst-4::gfp* expression were examined with fluorescent microscope at indicated time points. Representative images were shown. (**B**) Data from 2 independent experiments described in (**A**) were normalized to the average of day-2 adulthood. Data were analyzed by two-tailed, paired student’s t-test (**, P<0.01). Error bars indicate standard deviation of the mean. (**C**) *daf-2* worms recovered from oxidative stress faster than wild-type controls. Age-matched worms expressing *gst-4::gfp* were raised at 20° C. *gst-4::gfp* expression was examined at indicated time points. Two independent experiments were performed. Data were normalized to the average of day-2 adulthood and analyzed by two-tailed, paired student’s t-test (*, P<0.05. ***, P<0.001). Error bars indicate standard deviation of the mean. (**D**) L-carnitine did not further increase lifespan of *daf-2* *C. elegans*. Age-matched wild-type or *daf-2* worms were raised at 20° C throughout life with and without 10 μM L-carnitine supplement. Dead and live worms were counted every 2 or 3 days starting from day-10 of adulthood. Data from 2 experiments were pooled and analyzed by log-rank test (Supplementary Table 2). (**E**) L-carnitine did not further increase lifespan of *glp-1* worms. Age-matched wild-type or *glp-1* worms were raised at 25° C from L1 to L4 stage and then maintained at 20° C throughout life. 10 μM L-carnitine supplement was added starting from L1 stage. Dead and live worms were counted every 2 or 3 days starting from day-10 of adulthood. Data from 2 experiments were pooled and analyzed by log-rank test (Supplementary Table 2).

The requirement for DAF-16 and SKN-1 for lifespan extension by L-carnitine prompted us to test the role of L-carnitine in long-lived mutants *glp-1* and *daf-2*. These animals, similar to carnitine-treated animals, require both DAF-16 and SKN-1 for their long lifespan [36, 51]. First, we asked if *glp-1(e2144)* and *daf-2(e2370)* mutants would recover from oxidative stress better than wild-type controls. As reported earlier [35], *gst-4::gfp* expression was much higher in *glp-1* mutant reaching adulthood (Figure 4A). To evaluate the recovery, we normalized *gst-4::gfp* expression levels at day-2 adulthood and examined the speed of decline in GFP intensity. Indeed, *glp-1(e2144)* showed a faster decline in *gst-4::gfp* expression than controls (Figure 4A, 4B). Similar results were obtained for *daf-2(e2370)* mutants (Figure 4B). We then asked if such improved recovery from OSR could lead to enhanced clearance of endogenous ROS levels. Indeed, the experiments showed that both *glp-1(e2144)* and *daf-2(e2370)* mutant worms accumulated ROS slower than wild-type control (Figure 4C), suggesting that the speed to recover from OSR plays an important role in aging.

Second, we tested if L-carnitine could further increase the lifespan of *glp-1* and *daf-2* mutants. In several biological repeats, we found that L-carnitine could no longer increase lifespan of *glp-1(e2144)* and *daf-2(e2370)* mutant (Figure 4D, 4E). These results suggest that the activation of DAF-16 and SKN-1 in *glp-1* and *daf-2* mutants dominate the effect of L-carnitine, making these mutants no longer sensitive to L-carnitine.

### A potential carnitine transporter T08B1.1 promotes oxidative stress recovery and increases lifespan in *C. elegans*

Next, we investigated the mechanisms by taking advantage of the long-lived *glp-1* and *daf-2* mutants. We focused on genes that are upregulated by both mutants.

**Figure 5:**
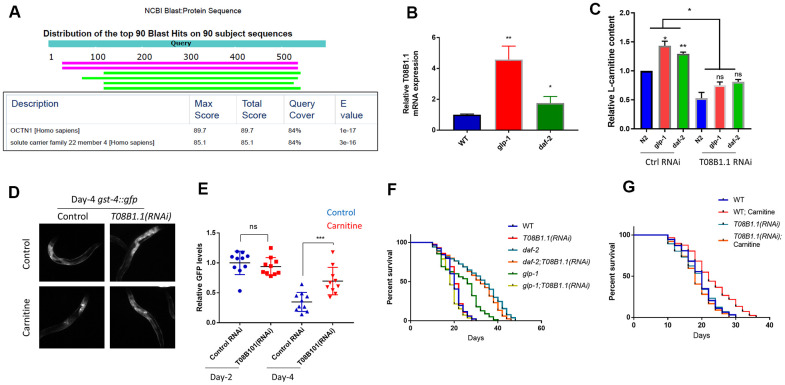
**A potential carnitine transporter T08B1.1 promotes oxidative stress recovery and increases lifespan in *C. elegans*.** (**A**) T08B1.1 is homologous to human carnitine transporter OCTN1. Protein sequence of T08B1.1 was used for blasting search with NCBI website with default setting for homologous sequences in human non-redundant protein sequences database. Table shows blasting results of the top 2 matched sequences. (**B**) T08B1.1 expression was elevated in *glp-1* and *daf-2* mutant worms. Age-matched *glp-1* worms were raised at 25° C from L1 to L4 stage then changed to 20° C. Age-matched *daf-2* worms were kept at 20° C. mRNAs were extracted at day-1 of adulthood and RT-qPCR was performed. Data from 3 experiments (each with 3 replicates) were analyzed with two-tailed, paired student’s t-test (*, P<0.05. **, P<0.01). Error bars indicate standard deviation of the mean. (**C**) RNAi knocking down of T08B1.1 decreased L-carnitine content in *C. elegans*. Age-synchronized L1 worms were raised on NG medium supplemented with10 μM L-carnitine until day-1 adulthood. Worms were homogenized and relative L-carnitine content measured with L-carnitine Assay kit. Data from 2 experiments were analyzed with two-tailed, paired student’s t-test (ns, not significant, *P<0.05, **P<0.01). Error bars indicate standard deviation of the mean. (**D**) RNAi knocking down of T08B1.1 prevented L-carnitine from decreasing oxidative marker *gst-4::gfp*. Age-synchronized worms expressing *gst-4::gfp* were raised on NG medium supplemented with10 μM L-carnitine until day-4 adulthood. Worms were imaged for GFP levels. Shown are representative images from day-4 adulthood. (**E**) Quantification of day-2 and day-4 data from experiments shown in (**D**). Ten images from 2 experiments were quantified by ImageJ and analyzed with two-tailed, unpaired student’s t-test (***, P<0.001). Error bars indicate standard deviation of the mean. (**F**) RNAi knocking down of T08B1.1 decreased *glp-1* lifespan but not that of wild-type or *daf-2* mutant. *glp-1* worms were raised at 25° C from L1 to L4 stage and then changed to 20° C. WT and *daf-2* worms were kept at 20° C. Dead and live worms were counted every 2 or 3 days starting from day-10 of adulthood. Data from 2 experiments (n>120 for each curve) were analyzed by log-rank test (Supplementary Table 3). (**G**) RNAi knocking down of T08B1.1 prevented L-carnitine from extending lifespan. Age-matched N2 wild-type worms were kept at 20° C throughout life. Dead and live worms were counted every 2 or 3 days starting from day-10 of adulthood. Data from 2 experiments (n>120 for each curve) were analyzed by log-rank test (Supplementary Table 4).

One such genes, T08B1.1, encoding a potential cation transporter, showed similarity to the carnitine transporter gene OCTN1 in humans (Figure 5A). Multiple sequence alignment of T08B1.1 with OCTN1 proteins in human, chicken and *Drosophila* showed 3 conserved protein domains (Supplementary Figure 2), despite moderate similarity at the primary sequence. We confirmed that T08B1.1 mRNA was upregulated in *glp-1* and *daf-2* mutants by using qPCR (Figure 5B). We tested if T08B1.1 protein could mediate carnitine transport by RNAi knockdown. Animals were cultured in the presence of 10 μM L-carnitine with or without T08B1.1 RNAi bacteria. By using the L-carnitine Assay Kit from Abcam, we showed that *glp-1* and *daf-2* mutants have higher carnitine content than WT controls after feeding L-carnitine for 48 hours from L1 stage (Figure 5C). L-carnitine levels were all reduced after T08B1.1 knockdown, suggesting T08B1.1 is a potential carnitine transporter.

We continued to ask if T08B1.1 would be required for recovery from oxidative stress during normal aging. To this aim, we fed age-matched L1 *C. elegans* expressing *gst-4:gfp* with T08B1.1 RNAi bacteria on NGM plate in the absence or presence of 10 μM L-carnitine and checked GFP levels on day-4 of adulthood. Consistent with previous results, L-carnitine significantly reduced the *gst-4::gfp* expression, however, knocking down T08B1.1 reversed *gst-4::gfp* reduction (Figure 5D, 5E), confirming the role of T08B1.1 in oxidative stress recovery. Interestingly, T08B1.1 knockdown decreased the lifespan of *glp-1* mutant but did not affect the lifespan of *daf-2* mutant (Figure 5F). Consistently, T08B1.1 knockdown also blocked L-carnitine to extend lifespan, supporting the role of T08B1.1 in carnitine transport (Figure 5G).

## DISCUSSION

In this study, we found that L-carnitine could facilitate the recovery from oxidative stress, but did not affect the induction of OSR. L-carnitine delayed ROS accumulation during aging, reduced amyloid protein aggregation and prolonged lifespan. We also found that T08B1.1 gene, encoding a potential carnitine transporter, is required for efficient carnitine uptake and lifespan extension in worms treated with L-carnitine and germline mutant (*glp-1*) mutant. Together, by using the genetically tractable model, we have gained significant insights into the functions of L-carnitine on aging.

The effect of L-carnitine on oxidative stress recovery is unprecedented. L-carnitine is derived from lysine and methionine and such special structure could directly scavenge free radicals [1, 2]. Previous studies have shown that L-carnitine functions to reduce oxidative stress in mice, rat and humans [21, 52]. Among many other proposed mechanisms such as PI3K, AKT, ERK pathways [53, 54], the antioxidant effect of L-carnitine has been attributed to its role in activating Nrf2-dependent transcription [55–57]. However, we show that carnitine does not activate Nrf2 homolog SKN-1 in *C. elegans*. Rather, L-carnitine serves to facilitate the recovery from oxidative stress by mitigating SKN-1-mediated stress response. Therefore, L-carnitine likely functions through a feedback mechanism to control SKN-1 and oxidative stress. These results contrast with previous thoughts, opening new windows for further investigations into the underlying mechanisms. In addition, there are many antioxidative proteins other than SKN-1 and DAF-16 that could play more important roles in mediating L-carnitine’s effect on lifespan, which could be readily revealed by a genome-wide RNAi screen for suppressors of either markers (for example *gst-4::gfp*) or lifespan.

Our study has shown a direct role of L-carnitine in lifespan extension. L-carnitine has been shown to improve multiple aging phenotypes, including high blood lipid, high blood glucose, neurodegeneration and weak cardiac function [21, 58–60]. But its role in lifespan remains poorly studied. Only one study has been reported showing that acetyl-L-carnitine can extend the chronological lifespan in the budding yeast [27, 28]. By taking advantage of the genetically tractable model organism *C. elegans*, we show that L-carnitine was able to extend lifespan in worms, which was mediated through the potential carnitine transporter T08B1.1. Confirming the role of L-carnitine in lifespan regulation, we show that T08B1.1 is elevated in long-lived *daf-2* and *glp-1* mutants and required for *glp-1* mutant to live long. T08B1.1 knockdown does not decrease lifespan of *daf-2* mutant, likely due to the fact that the *daf-2(1370)* mutant relies less on SKN-1-mediated oxidative stress response as shown before [36]. Alternatively, the constitutive activation of SKN-1 in *daf-2* mutant could dominate the L-carnitine signaling, making the mutant insensitive to T08B1.1 RNAi knockdown. Our study has established *C. elegans* as a model for mechanistic study of L-carnitine’s effect on delaying aging, which could boost the discovery of new strategies against aging and related disease.

## MATERIALS AND METHODS

### Medium and bacteria and *C. elegans* strains

Medium used in this study were liquid LB medium for culturing bacterial food OP-50. For culturing RNAi bacteria HT115 bearing a plasmid, 50 ug/mL of Ampicillin was added to liquid LB medium. Nematode grow medium (NGM) were prepared as shown in [61]. *C. elegans* were raised at 20° C on NGM agar plates seeded with OP-50 bacteria. Worms were maintained by transferring 10 L1 larva to new plate every 3 day. Strains used in this study were: WT strain is N2 (Bristol), CL2006: dvIs2 [pCL12(unc-54/human Abeta peptide 1-42 minigene) + pRF4], *daf-16(mu86) I*, *daf-2(e1370) III*, *dvIs20[pAF15(gst-4::GFP::NLS)], glp-1(e2144) III*, *daf-2(e1370) III; dvIs20[pAF15(gst-4::GFP::NLS)]*, *glp-1(e2144) III*; *dvIs20[pAF15(gst-4::GFP::NLS)]*. *glp-1(e2144) III* mutant worms were phenotypically wild-type at 20º C and raised at 25° C from L1 to L4 stage to inactivate *GLP-1* gene product to deplete germline.

### Drug treatment

L-carnitine was prepared in 1 mM stock solution and added to the NGM plate to the final concentration of 100 μM at least 12 hours before use. 10 day-1 adult worms were transferred to the plate for egg laying for 2 hours. Age-matched eggs were then allowed to hatch. For paraquat treatment, 10 mM paraquat stock solution was added to the NGM plate to the final concentration of 1 mM at least 12 hours before use. Worms at L4/young adult stage were transferred to paraquat plates for 24 hours and transferred back to paraquat-free NGM plate for recovery. Juglone was added similarly as paraquat except that 300 μM final concentration was used.

### ROS detection

To detect ROS levels by using microscope, animals were stained with fluorescent dye Dihydroethidium (DHE). Specifically, worms raised on agar plates were washed with M9 buffer and then incubated in M9 buffer containing 3 μM DHE for 30 min. Worms were washed extensively in M9 buffer before the ROS signal was imaged with fluorescence microscope. Fluorescence intensity was quantified by using ImageJ software.

### Western blot of protein aggregates

Equal number of Aβ-expressing worms (CL2006) at L1 stage were raised to young adult stage and treated as indicated. Worms were washed from plates with ice-cold M9 buffer to remove bacteria. Worms were then sonicated in 1X RIPA Buffer (20 mM Tris-HCl pH 7.5. 150 mM NaCl, 1 mM Na2EDTA. 1 mM EGTA. 1% NP-40) then centrifuged at 18,400Xg for 20 min at 4° C. The supernatant is soluble fraction and the pellet is insoluble fraction. The insoluble pellet was washed 3 times with RIPA buffer then resuspended in 75 ml denaturing buffer (8 M Urea, 2% SDS, 50 mM DTT, 50 mM Tris pH 8). Protein samples from soluble and insoluble factions were boiled in SDS loading buffer and separated by SDS-PAGE and total protein were transferred to PVDF membrane for western blot analysis with anti-Aβ antibody (Abcam, ab10148) and β-actin (Abcam, ab14128).

### Aβ-induced paralysis assay

CL2006 worms expressing human Aβ(1-42) in body-wall muscles were allowed to lay eggs to new plates for 2 hours. Age-matched progenies were allowed to grow on NGM medium plates at 25° C. 50 μM FUDR (5-Fluoro-2′-deoxyuridine) was added at L4/young adult stage to inhibit reproduction. Worms were gradually paralyzed during adulthood. Worms that failed to move forward or backward when touched by a platinum wire were scored as “paralyzed”. Worms were scored for paralysis at the indicated time points starting from day-1 of adulthood.

### RNAi knockdown

RNAi knockdown was carried out by feeding worms with bacteria expressing double strain RNA (dsRNA) on NGM agar plate. All RNAi bacteria were from the Ahringer RNAi library [62]. To prepare the RNAi agar plate, RNAi bacteria were cultured at 37° C overnight and sub-cultured to early log phase at OD600=0.4. 100 ul of bacteria were added directly on NGM plate agar plate containing 50ug/ml Ampicillin and 1mM Isopropyl β-D-1-thiogalactopyranoside (IPTG) to induce dsRNA expression. For RNAi knockdown, 10 day-1 adult worms were transferred to the RNAi plate for egg laying for 2 hours. Age-matched eggs were then allowed to hatch and grow on RNAi bacteria plate. 50 μM FUDR (5-Fluoro-2′-deoxyuridine) was added at L4/young adult stage to inhibit reproduction.

### Lifespan assay

For lifespan assay in *C. elegans*, age-matched L1 worms were raised on either OP-50 or RNAi bacteria plate until L4/young adult stage, then 50 μM FUDR (5-Fluoro-2′-deoxyuridine) was added to inhibit reproduction. Worms may be transferred to new plates as required. The number of live and dead worms was recorded every 2 or 3 days starting from day 10. Exploded and bagged worms and worms with protruding vulva were censored. Death was defined as lack of any visible movement for 5 seconds after touching the tail and head with a platinum wire.

### Real time quantitative PCR

Age- matched worms were washed from NGM plates with ice-cold M9 buffer. Worms were washed 2 more times by centrifugation to remove residual bacteria. Total mRNA was extracted with TRIzol™ Plus RNA Purification Kit (ThermoFisher Scientific) by following manufacture’s protocol. mRNA was reverse-transcribed using One-step RT-qPCR kits (Takara). Quantitative PCR was performed using SYBR Green 2X Mater Mix (Applied Biosystems). Gene expression levels were normalized to actin (*ACT-1*). Primers were designed by Roche Universal Probe Library System Assay Design. Primers for T08B1.1 are: 5’-cgaagttattatggctggatcttc-3’ and 5’-tttgacgtccaaaatggtca-3’. Primers for ACT-1 are: 5’-tcggtatgggacagaaggac-3’ and 5’-catcccagttggtgacgata-3’.

### Carnitine content measurement

Worms were grown on NGM medium plate containing 100 μM L-carnitine from hatching. On the day of experiment, worms were washed extensively with M9 buffer to remove bacteria and L-carnitine. Worms were then homogenized by sonication. Worm lysate were centrifuged at 18,400Xg for 20 min at 4° C. L-carnitine levels in the lysate were detected with L-carnitine Assay Kit (Abcam) by following manufacturer’s protocol. Protein concentration was measured by using BCA protein Assay Kit (Abcam) and served as input control.

## Supplementary Material

Supplementary Figures

Supplementary Tables
