# Supplementary material for: Carnitine promotes recovery from oxidative stress and extends lifespan in C. elegans
Source: Aging (Albany NY). 2020 Dec 3;13(1):813–30. doi: 10.18632/aging.202187 (PMC7835055; doi:10.18632/aging.202187)
Supplement: Supplementary Figures [file aging-13-202187-s001.pdf]

## SUPPLEMENTARY FIGURES

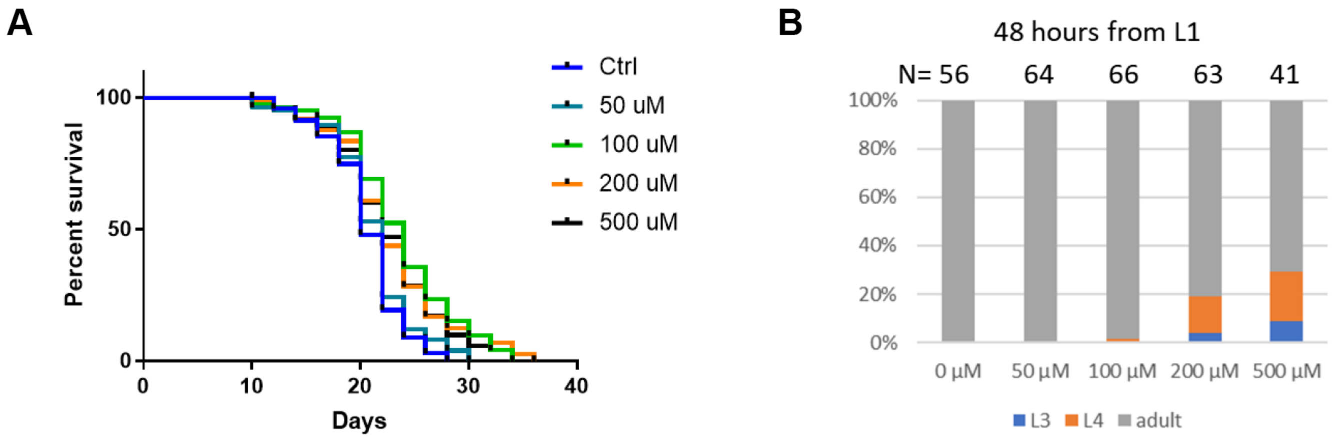


**Supplementary Figure 1.** (**A**) L-carnitine extends lifespan at various concentrations. Wild-type *C. elegans* were synchronized at L1 larvae stage and raised on NG medium supplemented with or without 10 µM L-carnitine. 50 µM FUDR was added to prevent reproduction. Dead and viable worms were counted every 2 or 3 days starting from day-10 of adulthood. Data were analyzed by log-rank test (Supplementary Table 5). (**B**) 200 and 500 µM L-carnitine cause developmental delay in some worms. Synchronized L1 worms in (**A**) were checked for development stage at L3, L4 and adult. Percentage of L3 and L4 in the whole population were shown in blue and orange, respectively.

A

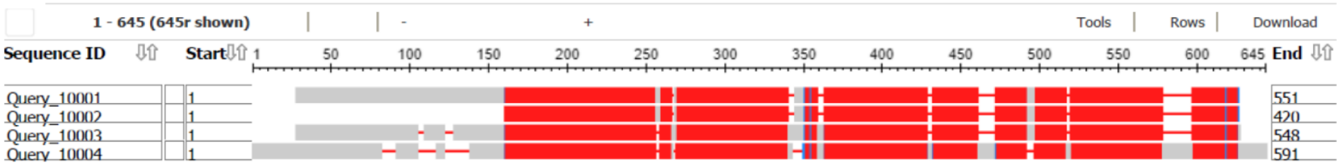


B

```
Query_10001  1    ----------------------------MRDYDEVIAFLGEWGPFQRLIFFLLSASIIPNGFNGMSVVFLAG-TPEHRCR  51
Query_10002       --------------------------------------------------------------------------------
Query_10003  1    ----------------------------M-GYDDVITHLGEFGPYQKRIYYLLCLPAIVCAFHKLAGVFLLA-KPDFRCA  50
Query_10004  1    msefeevelkqirptyakkrkekkdvvfYTNFEEVLQKIGAFGPYQIFCFIVILYASIEWAGNSTFMHLLGSfEPDWNCT  80

Query_10001  52   VPDAANLSSAWRNNSVPLRLRDGREvphsCSRYRLATIANFSalglepGRDVDLGQLEQESCLDGWEFSQDVYLSTVVTE  131
Query_10002       --------------------------------------------------------------------------------
Query_10003  51   LPYENGSIYELSPHLWNLSYPENER----CSYYDVDYTEEYL------NGSIPRSSNETKTC-SSYVYDRSKYLNSAVTE  119
Query_10004  81   LA---------NNQTVIITAPTHDD------------TCNFI----------------KQNCTNLAPVKQNLEFFSIVGQ  123

Query_10001  132  WNLVCEDNWKVPLTTSLFFVGVLLGSFVSGQLSDRFGRKNVLFATMAVQTGFSFLQIFSISWEMFTVLFVIVGMGQISNY  211
Query_10002  1    WNLVCEDDWKVPLTTSLFFVGVLIGSFISGQLSDRFGRKSILFLTMAVQTGFSFLQIFSTSWEMFTVLFLIVGMGQISNY  80
Query_10003  120  WNLVCSRSLLSATSDSLFMLGVLLGSLIFGQMSDKLGRKPTFFASLVLQLIFGVLAAVAPEYFSYTISRMIVGATTSGVF  199
Query_10004  124  FQLICDDSDKVEYIEVIMAGSSLIGSIIGGHMGDHFGRQTIFFTGELLIIITSMMCTAAQSWIAFSVIQGVNCFLYGVIE  203

Query_10001  212  VVAFILGTEILGKSVRIIFSTLGVCT--FFAVGYMLLPLFAYFIRDWRMLLLALTVPGVLCVPLWWFIPESPRWLISQRR  289
Query_10002  81   VVAFILGTEILGKSVRILFSTLGVCV--FFAIGYMLLPLFAYFIRDWRMLLLALTLPGLLCVPLWWIIPESPRWLISQGR  158
Query_10003  200  LVAYVIALEMVGSSYR---LFAGVAMQmFFSVGFMLTAGFAYFIHDWRWLQIAITLPGLLFLCYYWIIPESARWLLMKGR  276
Query_10004  204  TTSLTMMVEFTSNKFR---VIMVNAFQ--WPIAYMTIALIAWLTQGWQVYFVFLNLVSSPLAIGFMLFLESPRWLIARND  278

Query_10001  290  FREAEDIIQKAAKMNNIAVP-----AVIFDSVEELNPLK----QQKAFILDLFRTRNIAIMTIMSLLLWMLTSVGYFALS  360
Query_10002  159  YKEAEVIIRKAAKINNTPAP-----AMLFDAAEDSKPQQ----QQKAILLDLFRSRNIATITIMSLLLWFFTSVGYFGLS  229
Query_10003  277  KDEAFVIIEKAAKENKVEVPNEIyeQLVDEVAEKKKQDEMAASQPAATVFDLLRYPNLRRKTLLIFFDWFVNSGVYYGLS  356
Query_10004  279  LSEACEVLNDIAHQRWNNTKARF-------TTKDISAIHKQEKQGFYWFYHLFSTKRLAKQSCLQIISVLTYAMVSNTYL  351

Query_10001  361  LDAPNLHGDAYLNCFLSALIEIPAYITAW---LLLRTLPRRYIIAAVLFWGGGVLLFIQLV-----------PVDYYFLS  426
Query_10002  230  LNTPNLHGDAYINCFLSAVIEVPAYIIAW---LLLRSLPRRYSISGTLVLGGGVVLFINLV-----------PTDLNVLS  295
Query_10003  357  WNTNNLGGNQLVNFMISGAVEIPGYTLLL---FTLNRWGRRSILCGTMMVAGISLLATIFV-----------PSDMNWLI  422
Query_10004  352  FTVAGLHDSPIISTLIDGVLRLFIPIIIVifdLMVPSFGRKIQFLGSLVIEGILFGVVIALvatgtctydskAVNILVIV  431

Query_10001  427  IGLVMLGKFGITSAFSMLYVFTAELYPTLVRNMAVGV---TSTASRVGSIIAPYFVYLGAYNRMLPYIVMGSLTVLIGIL  503
Query_10002  296  VCLVMLGKFGITAAFSMLYVFNVELYPTLVRNMAVGA---TSTASRLGSIIAPYFVYLGAYDRYLPYIVMGSLTVLIGIL  372
Query_10003  423  VACAMIGKLAITSSYGTIYIFSAEQFPTVVRNVGLGA---SSMVARVGGILAPYLKLLGEIWRPLPLIICGALSLTAGLL  499
Query_10004  432  TTMINDCIFWIN-----IVQITTQRYPTVIRCVAFGFlhsFRHIGAIIGFLILKPLLTSTWPVGAFVIPEAMIVLTILVG  506

Query_10001  504  TLFFPESLGMTLPETLEQ------------------MQKVKWFRSGKKTRDSMETEENPKVLITAF--------------  551
Query_10002  373  TLFLPESYGSALPESFEQ------------------MMKVKCFRNGQQTTGIRNSKESPKTLITTL--------------  420
Query_10003  500  SLLLPETLNKPMPETIED------------------GENFGKKPAPQETAEEGGTQELSGMLNGKSG-------------  548
Query_10004  507  FFFQPETKGKALMDQMVEanygrlenelpralmrlaaGHRVDQSETRQQHRKDMESAKAAELMGKKVSspwvfkekdsgp  586
```

**Supplementary Figure 2. Alignment of carnitine transporter OCTN1 and homologs in different species.** Human (OCTN1, Query_10001), Chicken (OCTN1, Query_10002), *Drosophila* (NP_001262908.1, Query_10003) and *C. elegans* (T08B1.1, Query_10004) were aligned by Multiple Alignment tools from NCBI website. Conserved domains (**A**) and corresponding amino acids (**B**) were shown in red.
