# Supplementary material for: Carnitine promotes recovery from oxidative stress and extends lifespan in C. elegans
Source: Aging (Albany NY). 2020 Dec 3;13(1):813–30. doi: 10.18632/aging.202187 (PMC7835055; doi:10.18632/aging.202187)
Supplement: Supplementary Tables [file aging-13-202187-s002.pdf]

SUPPLEMENTARY TABLES

Supplementary Table 1. Lifespan data and statistics to Figure 3E–3G.

Treatment	Number of worms	Death	censored	Median lifespan (days)	Compare to	P values
Experiment 1						
WT Ctrl	88	81	7	20		
L-Carnitine	95	88	7	22	WT	0.0022
skn-1(RNAi)	85	82	3	18		
skn-1(RNAi), L-Carnitine	76	72	4	18	*skn-1(RNAi)*	0.4645
daf-16(mu86)	85	82	3	18		
daf-16(mu86), L-Carnitine	72	68	4	18	*daf-16(mu86)*	0.2214
Experiment 2						
WT Ctrl	66	61	5	20		
L-Carnitine	55	50	5	22	WT	0.0152
skn-1(RNAi)	48	48	0	18		
skn-1(RNAi), L-Carnitine	53	48	5	18	*skn-1(RNAi)*	0.5114
daf-16(mu86)	53	50	3	18		
daf-16(mu86), L-Carnitine	51	50	1	16	*daf-16(mu86)*	0.2513

Supplementary Table 2. Lifespan data and statistics to Figure 4D, 4E.

Treatment	Number of worms	Death	censored	Median lifespan (days)	Compare to	P values
Experiment 1						
WT Ctrl	92	87	5	20		
L-Carnitine	96	90	6	22	WT	0.0003
daf-2(e2370)	83	75	8	36		
daf-2(e2370), L-Carnitine	88	80	8	36	daf-2(e2370)	0.8026
glp-1(e2144)	83	80	3	28		
glp-1(e2144), L-Carnitine	80	75	5	26	glp-1(e2144)	0.3893
Experiment 2						
WT Ctrl	75	67	8	20		
L-Carnitine	75	69	6	22	WT	0.0014
daf-2(e2370)	59	49	10	36		
daf-2(e2370), L-Carnitine	69	60	9	34	daf-2(e2370)	0.6032
glp-1(e2144)	62	60	2	28		
glp-1(e2144), L-Carnitine	57	50	7	26	glp-1(e2144)	0.2398

Supplementary Table 3. Lifespan data and statistics to Figure 5F.

Treatment	Number of worms	Death	censored	Median lifespan (days)	Compare to	P values
Experiment 1						
WT Ctrl	92	87	5	20		
T08B1.1(RNAi)	86	83	3	20	WT	0.6642
daf-2(e2370)	84	78	7	34		
daf-2(e2370), T08B1.1(RNAi)	87	83	4	32	*daf-2(e2370)*	0.0991
glp-1(e2144)	87	79	8	26		
glp-1(e2144), T08B1.1(RNAi)	122	112	10	18	*glp-1(e2144)*	<0.0001
Experiment 2						
WT Ctrl	69	62	7	20		
T08B1.1(RNAi)	62	56	6	22	WT	0.9635
daf-2(e2370)	65	59	6	32		
daf-2(e2370), T08B1.1(RNAi)	64	62	2	32	*daf-2(e2370)*	0.4459
glp-1(e2144)	68	62	6	26		
glp-1(e2144), T08B1.1(RNAi)	66	59	7	22	*glp-1(e2144)*	0.0022

Supplementary Table 4. Lifespan data and statistics to Figure 5G.

Treatment	Number of worms	Death	censored	Median lifespan (days)	Compare to	P values
Experiment 1						
WT Ctrl		64	10	20		
T08B1.1(RNAi)		56	0	20	WT	0.8960
L-Carnitine		83	9	22		
L-Carnitine, *T08B1.1(RNAi)*		89	0	18	L-Carnitine	<0.0001
Experiment 2						
WT Ctrl		54	8	20		0.1370
T08B1.1(RNAi)		73	6	20	WT	
L-Carnitine		46	10	22		
L-Carnitine, *T08B1.1(RNAi)*		72	11	20	L-Carnitine	0.0002

Supplementary Table 5. Lifespan data and statistics to Supplementary Figure 1A.

L-carnitine concentration (µM)	Number of worms	Death	censored	Median lifespan (days)	Compare to	P values
0	75	67	8	20		
50	81	74	7	22	0 µM	0.2485
100	82	73	9	24	0 µM	<0.0001
200	79	71	8	22	0 µM	0.0008
500	76	70	6	22	0 µM	0.0009
